# Cervicobrachialgia in a female in her fourth decade of life: Have Eagle’s syndrome and thoracic outlet syndrome been considered?

**DOI:** 10.23938/ASSN.1146

**Published:** 2025-11-03

**Authors:** Vicente Martín Moreno, Amanda Martín Fernández, Jorge Hurtado Gallar, Cristina Angulo García, Marina Pérez Blanco

**Affiliations:** 1 Servicio Madrileño de Salud Centro de Salud de Orcasitas Madrid España; 2 Instituto de Investigación Sanitaria Hospital 12 de Octubre (i+12) Madrid España; 3 Polibea Concierto SL Madrid España

**Keywords:** Eagle’s syndrome, Thoracic outlet syndrome, Ankle-brachial index, Cervicalgia, Paresthesia, Síndrome de Eagle, Síndrome del desfiladero torácico, Índice tobillo brazo, Cervicalgia, Parestesia

## Abstract

Cervicobrachialgia is a common reason for consultation among women in the fourth and fifth decades of life. However, the diagnostic approach is often incomplete, leading to delays in both diagnosis and treatment. We report the case of a 44-year-old woman presenting with left craniocervical pain of several years’ duration, radiating to the left arm and the pectoral and dorsal regions, accompanied by paresthesia. Physical examination revealed findings compatible with Eagle’s syndrome (tonsillar fossa pain) and thoracic outlet syndrome (TOS) (positive Adson, Wright, and Roos tests). Assessment using the ankle-brachial index demonstrated vascular involvement, suggesting a potential new diagnostic approach for TOS. Cranial radiographs showed hyperdevelopment of both mastoid processes, while orthopantomography demonstrated elongation (>5 cm) of the left styloid process, which was confirmed by computed tomography that also revealed a duplicated C3 rib with supraclavicular course, confirming the coexistence of Eagle’s syndrome and TOS, an uncommon association in clinical practice.

## RESUMEN

La cervicobraquialgia es motivo frecuente de consulta en mujeres en la cuarta-quinta décadas de la vida, realizándose con frecuencia un abordaje incompleto, que retrasa el diagnóstico y tratamiento. Presentamos el caso clínico de una mujer de 44 años, con dolor craneocervical izquierdo de años de evolución, irradiado a brazo y zonas pectoral y dorsal izquierdas, con parestesias. La exploración mostró datos compatibles con los síndromes de Eagle (dolor en fosa tonsilar) y del desfiladero torácico. El estudio mediante el índice tobillo-brazo mostró compromiso vascular, proponiéndose un nuevo test para el diagnóstico de síndrome del desfiladero torácico. La radiología de cráneo mostró hiperdesarrollo de ambas apófisis mastoideas, y la ortopantomografía una elongación >5 cm de la apófisis estiloidea izquierda, confirmada mediante tomografía, observándose también una costilla C3 duplicada con recorrido supraclavicular, confirmando la coexistencia de los síndromes de Eagle y del desfiladero torácico, situación poco frecuente en la práctica clínica.

## INTRODUCTION

Several studies have established that one in five people experience chronic pain, and that one in four of these individuals are unaware of its cause^1^. Women are more likely to suffer chronic pain, with cervicobrachialgia being a particularly prevalent condition, especially among women in their fourth and fifth decades of life^1^. Clinical consultations arising from this nosological entity often involve an incomplete etiopathogenic approach, leading to repeated visits and medical consultations between departments over years, thereby delaying diagnosis and treatment.

Cervicobrachialgia can have multiple causes, one of which is Eagle’s syndrome - a relatively prevalent condition^2^ characterized by elongation of the styloid process and/or calcification of the stylohyoid ligament. These anatomical changes produce symptoms that allow differentiation between two clinical presentations, classic and vascular, which may coexist in the same patient. Eagle’s syndrome can lead to complications such as carotid artery dissection, transient ischemic attacks, and recurrent syncope^3^. Compression of the jugular vein may also result in intracranial hypertension, headache, pulsatile tinnitus, and dizziness. Additional symptoms can occur depending on the location and orientation of the styloid process^4^^,^^5^ and the presence of other cranio-cervical alterations or anatomical variations, which may represent further subtypes of Eagle’s syndrome^5^.

Regarding these anatomical variants, the mastoid process forms part of the temporal bone, which also houses important structures such as the carotid canal, the jugular foramen (internal jugular vein, cranial nerves IX, X, and XI, and the posterior meningeal artery), and the internal auditory canal (cranial nerves VII and VIII, and the labyrinthine artery). Mastoid hypertrophy may produce compression within these canals or contribute to insertional anomalies of the sternocleidomastoid muscle^4^.

The complexity of the cranio-cervical and shoulder regions further predisposes to disorders causing pain. In women aged 40-55 years, cervicoarthrosis, disc herniation-protrusions, and positional abnormalities of the cervical spine account for most cases of cervicobrachialgia^6^.

Thoracic outlet syndrome (TOS), also as known scalene syndrome, should also be considered among the differential diagnoses of cervicobrachialgia. TOS is a rare disease (Orphanet descriptor 97330) characterized by pain and paresthesia due to compression of the neurovascular bundle^7^, symptoms that typically worsen with movement of the affected arm. Three types of TOS have been described - neurogenic, venous, and arterial - with the neurogenic form accounting for approximately 95% of cases. However, mixed presentations may occur within the same patient^7^^,^^8^. TOS is likely underdiagnosed, usually presenting between the ages of 30 and 40 years^8^ and affecting women more frequently^7^.

Nevertheless, these diagnoses do not always account for the full clinical picture in patients with cervicobrachialgia^1^. In an unknown proportion of cases, the assigned diagnosis may not represent the true or sole cause of the patient’s pain^1^^,^^6^. Clinical practice demonstrates that a positive diagnostic test does not necessarily explain a patient’s symptoms^6^, yet clinicians often remain confined within this diagnostic framework, without considering alternative possibilities.

Here, we report the case of a 44-year-old woman with long-standing cervicobrachialgia and paresthesia, in whom Eagle’s syndrome, TOS, a mastoid process anatomical variant, and a duplicated and aberrant third dorsal rib coexist.

## CASE REPORT

A 44-year-old woman with anthropometric data of weight 42 kg, height 153 cm, and body mass index 17.94, without toxic habits (tobacco, alcohol, or other substances), and not engaged in paid employment, presented with a history of left lateral neck pain persisting for more than four years. She describes the pain as constant and incapacitating, rating it as 9 on the Visual Analog Scale.

The pain radiated in a fan-like distribution, producing left hemicrania extending from the ocular orbit and preauricular region to the occipital area, and it intensified with head and neck movements. Distally, the pain radiated to the left shoulder, spreading to the pectoral region and, laterally and dorsally, down the arm to the hand, and was associated with paresthesia.

Clinical examination suggested several possible etiologies, including Eagle’s syndrome and TOS.

*Eagle’s syndrome:* palpation of the tonsillar fossa through the skin elicited stabbing pain, radiating to the ear and occipital region, whereas direct palpation of the pharyngeal tonsillar fossa produced only mild discomfort. The patient had a previous diagnosis of bruxism and was using a night splint, without symptomatic improvement. Mobilization and compression of both temporomandibular joints did not reproduce the pain. Cervical lateralization and contralateral cephalic rotation provoked pain, limiting cervical mobility. Glossodynia, dysgeusia, and odynophagia were absent; however, the patient reported excessive salivation (frequent drooling) and a persistent retropharyngeal foreign-body sensation. Occasionally tinnitus was also present.

*TOS:* electromyography ruled out a neurogenic origin, showing no involvement of the brachial plexus or focal mononeuropathies, and no evidence of active denervation or motor unit loss in the left C3, C4, C5, C6, C7, and T1 myotomes. Sustained elevation of the left upper limb did not reveal block of the ulnar nerve or asymmetries compared with the contralateral side. The presence of arterial vascular compromise and its association with pain and paresthesia were evaluated using a novel method: the ankle-brachial index, obtained with a MESI® device (model MTABLET; MESI Ltd, Slovenia).

**Figure 1:**
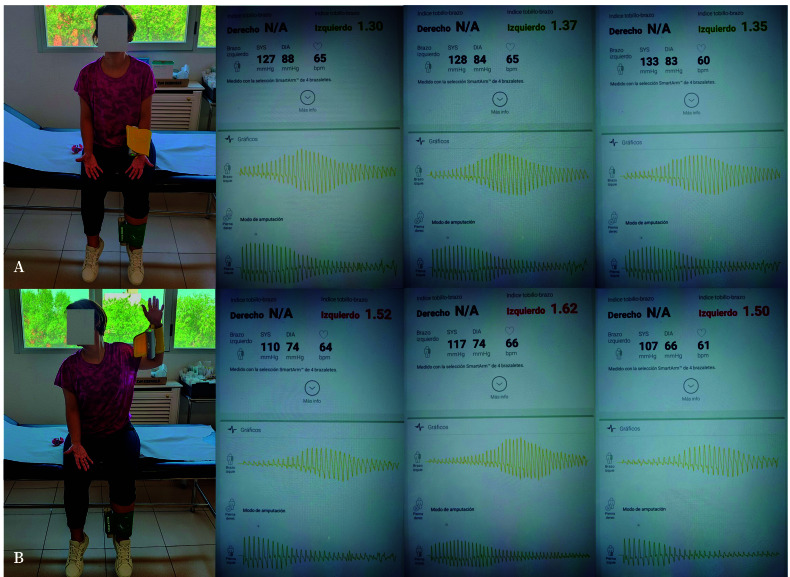
Registration of arterial involvement using the ankle-brachial index as a diagnostic method in thoracic outlet syndrome. **A.** Maneuver 1. **B.** Maneuver 2. Between Maneuvers 1 and 2, only the position of the left arm changes, allowing estimation or exclusion of vascular compromise when elevating the limb. Text and numerical values in red indicate abnormal parameters, while those in green indicate normal ones. The graphs correspond to the arterial pulse in the left arm (yellow) and the left leg (green). Between positions 1 and 2, a rightward shift of the pulse graph is observed in the left arm, which is not seen in the left leg.

The ABI is a non-invasive diagnostic test that measures differences in arterial pressures between limbs, and is routinely used to screen for peripheral arterial disease. Although conventionally designed for lower limb assessment, in this case to method was applied inversely to evaluate the upper limbs, which is technically feasible with current devices. Sensors were placed on the wrist (radial artery) and ankle (tibial artery); measurements were obtained in two positions (Fig. 1):

- *Maneuver 1:* In a seated position, the patient kept her arms parallel to the body, forearms supinated and resting on the thighs, palms facing upwards, fingers extended and relaxed (Fig. 1A).
- *Maneuver 2:* From position 1, the evaluated arm was flexed 90º at both the shoulder and elbow, with the palm facing forward and fingers extended and separated, while the head was rotated contralaterally (Fig. 1B).

In Maneuver 1, the ABI were 1.30, 1.37, and 1.35, with the pulse waveform showing a maximum peak centered or shifted to the left (Fig. 1). In Maneuver 2, pain and paresthesia were reproduced, and the ABI values increased to 1.52, 1.62, and 1.50. In six measurements, ABI could not be determined due to the absence of systolic and diastolic pressures and the test was interrupted on three occasions because of severe pain and paresthesia. The pulse waveform showed a leftward shift (Fig. 1).

The Adson, Eden, and Wright maneuvers were positive, producing attenuation or disappearance of the radial pulse. The Roos maneuver elicited pain and paresthesia in the left shoulder and arm within 5-6 seconds, symptoms that also reproduced during the Halsted and Morley tests.

Pain was likewise reproduced during the first Elvey maneuver (shoulders abducted at 90º, elbows and hands extended), and the second Elvey maneuver (same posture, with wrist dorsiflexion). In the third Elvey maneuver, performed from the second posture with right lateral flexion of the head, pain appeared in the shoulder and left cervical region. Even passive elevation of the arms triggered pain and paresthesia, and the left supraclavicular Tinel sign was positive. Palpation of the scalene triangle also provoked pain and paresthesia. No Gilliat-Sumner hands deformities (thenar and hypothenar atrophy, or interosseous muscle hypotrophy) were observed. The QuickDASH score was 67.1%, indicating marked functional limitation. Mean triple blood pressure was 123/75 mmHg in the right arm and 126/79 mmHg in the left. Physiotherapeutic assessment revealed an imbalance between diaphragmatic and scalene muscle activation.

**Figure 2:**
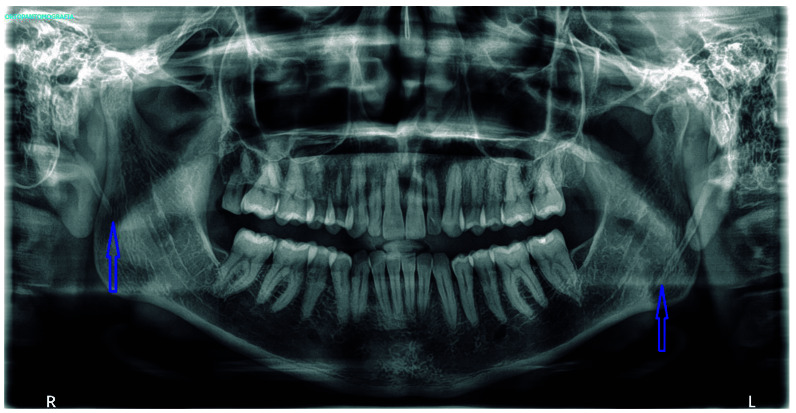
Orthopantomography. Elongated left styloid process (red arrow) compared to the right process (blue arrow).

Skull radiography demonstrated hyperdevelopment and pneumatization of both mastoid processes, which likely altered the insertion and course of the sternocleidomastoid muscles. Orthopantomography revealed elongation of the left styloid process exceeding 5 cm (Fig. 2).

**Figure 3:**
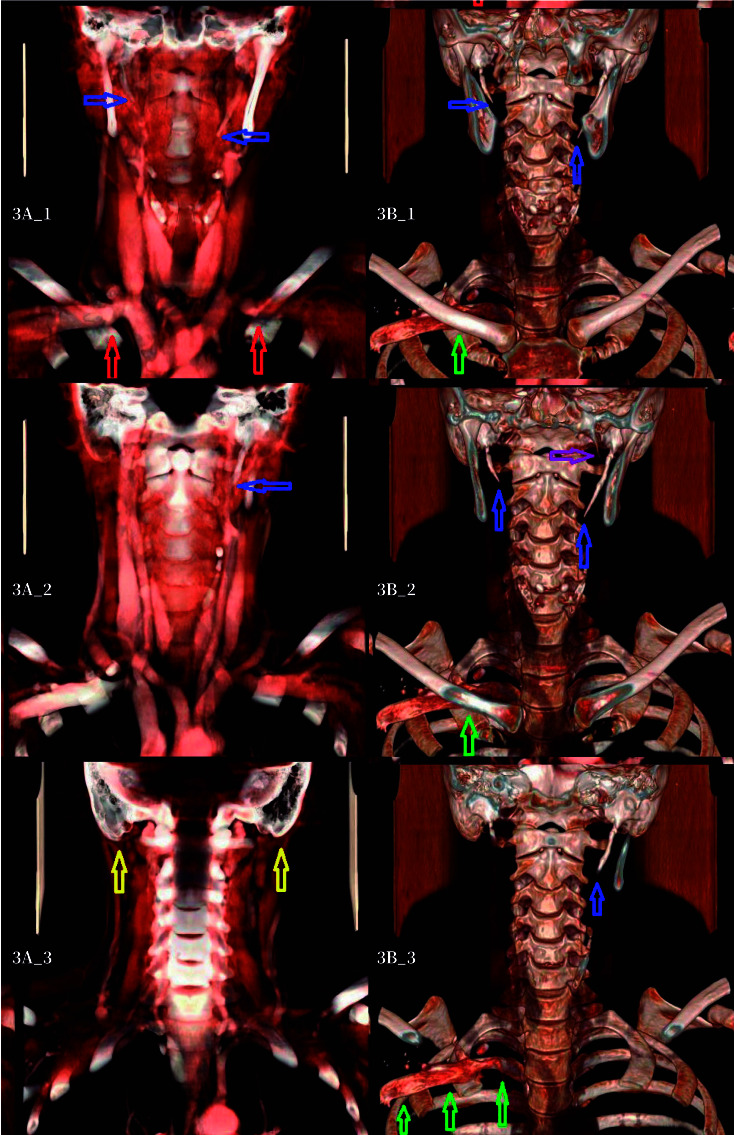
A. Cervical computed axial tomography, showing elongation of the left styloid process (blue arrows), vascular structures (red arrows: neurovascular bundle in the thoracic outlet), and bilateral mastoid hyperdevelopment with pneumatization (yellow arrows), with pneumatization, more pronounced on the left. B. Computed axial tomography showing elongation of the left styloid process (blue arrows) an elongated pyramidal of the palatine bone (purple arrow), and a duplicated right rib (green arrows).with aberrant anterior course located above the first rib and below the right clavicle.

These findings were confirmed on computed axial tomography (CT) performed (Fig. 3A), which additionally showed an elongated pyramidal process of the left palatine bone and duplicated C3 rib with a right supraclavicular trajectory (Fig. 3B).

Clinic, physical, and complementary findings (CT, ABI) confirmed the coexistence of Eagle’s Syndrome and arterial TOS, a rare association in clinical practice.

After the presumptive diagnosis was established, the patient was informed about the nature of her condition and the available treatment options. The anxiety associated with chronic pain and her concern that the pain signified a serious illness - leading to insomnia - was managed with mirtazapine 15 mg once daily at dinnertime. For chronic pain, celecoxib/tramadol 56 mg/44 mg was prescribed every 12 hours for one month, followed by etoricoxib 90 mg every 24 hours for one month, after symptomatic improvement. Acetaminophen 650 mg as needed was also maintained. Although no brachial plexus involvement was demonstrated, the neuropathic characteristics of the pain justified empirical treatment with pregabalin 50 mg every 12 hours for the first month, increased to 75 mg every 12 hours thereafter, to be continued until pain improvement or resolution. For episodes of acute pain, desketoprofen 25 mg every 8 hours was prescribed as needed.

Concurrently, a long-term physiotherapeutic program was indicated, focusing on achieving a balance between relative rest of the affected area and individualized strengthening and mobility exercises, as well as respiratory restraining to optimize diaphragmatic use and prevent compensatory overuse of the scalene muscles.

## DISCUSSION

Reality sometimes surpasses fiction, and this case is paradigmatic. As in the case of ageism, being a woman between 40 and 55 years of age often leads to the trivialization of symptoms, attributing them to a transient stage of life and/or to the medicalization of these symptoms without performing diagnostic studies. This delay in diagnosis has a direct impact on patients’ quality of life^7^.

The main contribution of this case is that a comprehensive clinical assessment allowed us to identify a complex combination of causes that justified the patient’s symptoms. A second contribution is the observation of an association between Eagle’s syndrome and TOS as a cause of cervicobrachialgia - an association not previously described. Finally, this case demonstrates that the ankle-brachial index can graphically record the reduction or absence of the pulse during the classic TOS maneuvers. In the presence of pain during these maneuvers, the ankle-brachial index could represents a novel diagnostic tool for TOS.

Regarding Eagle’s syndrome, the patient’s symptoms could be explained by stimulation of the internal carotid (causing parietal headache) and stylocarotid (causing facial pain) arteries^9^. A thorough physical examination and CT - the gold standard^1^^,^^3^^,^^9^ for diagnosis - generally confirm the condition, while orthopantomography and magnetic resonance imaging can also be useful^10^^,^^11^.

The patient also met the diagnostic criteria proposed by the Consortium for Outcomes Research and Education on Thoracic Outlet Syndrome^12^^,^^13^. These criteria include tests with sensitivities and specificities below 90%, some of which involve a degree of subjectivity (e.g., assessment of radial pulse attenuation or disappearance). However, when several tests yield positive results, the probability of a correct diagnosis increases.

In this case, Maneuver 2 (Fig. 1) produced pain and paresthesia, with ABI values above 1.4 - consistent with arterial compromise -, and in several measurements, pulse loss made ABI values unmeasurable. Therefore, this maneuver with ABI recordings is proposed as a new diagnostic test for TOS (the Martin-ABI test)^14^. As this finding derives from a single case, further validation in larger studies is required.

Other anatomical variation may also have contributed to the patient’s symptoms^4^.

The differential diagnosis rules out Ernest’s syndrome, which affects the stylomandibular ligament and presents clinical overlap with Eagle’s syndrome, though the presence of calcified elongation differentiates the two^15^. Collet-Sicard syndrome was also excluded, despite the presence of mastoid hyperdevelopment and elongation of the pyramidal process of the palatine bone. Although this syndrome can cause cervicobrachialgia, our patient exhibited no cranial nerve involvement. Likewise, there were no findings suggestive of spinal accessory nerve involvement.

Initial management of both syndromes was conservative^8^^,^^11^. Rehabilitation and physical therapy play a crucial role in TOS. Neurodynamic exercises improve muscular tone and range of motion, reducing pain. Their simplicity facilitates adherence, allowing the patient to continue them at home, which - as observed in this case - can enhance quality of life. Lifestyle modifications were also recommended, such as avoiding sleeping with the arms above the head or performing repetitive movements involving the arm or neck.

Before the suspected diagnosis, the patient had undergone several four-year treatment cycles with first-line analgesics (acetaminophen 650 mg, naproxen 550 mg, desketoprofen 25 mg) and one-month cycles of cyclobenzaprine hydrochloride 10 mg or duloxetine 30 mg, with limited or no benefit. After the diagnosis, pain intensity, its occurrence in daily activities, functional limitations, and impact on quality of life justified a multimodal pharmacological approach^8^^,^^11^. Empirical treatment with pregabalin proved effective in clinical improvement - possibly due to pleiotropic effects beyond analgesia - while also adding anxiety control and sleep, in combination with mirtazapine. Mirtazapine was maintained for three months and discontinued once anxiety and sleep disturbances resolved. Three months after initiating pregabalin, the patient’s improvement allowed progressive tapering of other medications. Currently, she remains on pregabalin 50 mg every 12 hours and acetaminophen 650 mg as needed.

Women are more likely to experience chronic pain and generally have a lower pain threshold, which adversely affects quality of life. Chronic pain also induces stress and anxiety, heightening pain sensitivity. This clinical case underscores the need to address chronic pain in women over 40 from a holistic perspective - one that includes a thorough medical history and physical examination to determine which patients require further testing. Trivializing chronic pain by attributing it solely to menopausal hormonal changes can delay diagnosis and deprive patients of targeted treatment. Furthermore, informing patients of the cause of their pain may itself serve as an initial therapeutic measure, one often undervalued by clinicians. Although rare, both Eagle’s syndrome and TOS should be considered in the differential diagnosis of cervicobrachialgia and pain syndromes involving the craniocervical region.

## Data Availability

Data are available under request to corresponding author.
